# Mozambique public investment in the water and sanitation sector and the targets of SDG6

**DOI:** 10.14324/111.444/ucloe.000067

**Published:** 2024-01-23

**Authors:** Manuel Salvador da Conceição Rebelo

**Affiliations:** 1 Faculty of Earth Science and Environment, Pedagogical University of Maputo, Maputo, Mozambique

**Keywords:** Mozambique, SDG6, access to water and sanitation, public investment

## Abstract

Many developing countries may not reach the targets of providing access to safe water sources and sanitation services for all by 2030. Census data from Mozambique show that the country’s population is one of fastest growing in the Sub-Saharan region. Between the 2007 and 2017 censuses more than seven million people were added to the total population. By 2030 about 11 million people will be added to Mozambique’s population. This will pose a huge challenge for the water and sanitation services. Access to these services is a fundamental requirement for the wellbeing of individuals and the development of nations. The last data from a Survey on Mozambique Family Budgets (IOF 2019/20), show that 55.7% of a total population has access to safe water sources. In contrast, access to sanitation services (31% of the population), has not kept pace with the progress made in water access. In this study, based on data from the General State Account of Mozambique, which includes the description of the annual investment made by the government and using the results of the Family Budget Surveys, it can be seen that if the average percentage values of public investment of 2009 to 2021 are the same in the following years as regards the water and sanitation sector, Mozambique will not reach the Sustainable Development Goals 6 targets in 2030.

## Introduction

Access to safe water sources and sanitation services is a fundamental requirement for the wellbeing of individuals [1] and for the economic growth of nations in general [2]. The absence or reduced access to these services contributes to the spread of pathogens that cause different types of disease [3], especially in the urban informal settlements that have developed as a result of unplanned urbanisation process [4].

Ensuring universal availability and sustainable management of safe water and sanitation services by 2030 is a Sustainable Development Goal (SDG). The SDG6 were adopted by the United Nations (UN) in 2015 [5,6]. The UN considers that achieving this goal requires large investments in adequate infrastructure, mainly in developing countries, where there is a significant historical delay in this field.

The progress made in accessing safe water sources at the global level is evident, inspiring and can help mobilise more and new financing. Data show that 91% of the global population has access to a source of safe drinking water [7]. On the contrary, access to sanitation services is advancing but at a slow pace, that is, 3.6 billion people do not have access to sanitation services and 8% of the total defecates in open spaces and a large part of this percentage are from the Sub-Saharan Africa region [2]. There are also substantial differences in access to these services between urban and rural areas.

Although the coverage rate does not capture elements such as quality and delivery of services, Mozambique has made little progress in access to water and sanitation compared to other African countries with similar incomes [4]. The highest rates of water access and sanitation services are in urban areas. The latest Survey on Mozambique Family Budgets (IOF 2019/20) shows that a little more than half of the population (55.7%) has access to a source of safe water for consumption and less than half (31%) use safe sanitation services, such as the use of sewage networks and other one-site sanitations systems (non-flush toilets, improved latrines and improved traditional latrines). In Mozambique the latrine is an outside toilet built of wood, grass and other local materials freely available in rural areas, but there is also a cement block version of this type of toilet found in city slums. This is a characteristic, which according to Minh [1] is commonplace in developing countries.

Access to safe water and sanitation services is determined by the investment that is made in infrastructure [3]. For example, relatively recent data indicated that the total world population using improved sanitation infrastructure in 2017 was 68% [7] and this is a progress resulting from investment in infrastructure.

There is an immediate positive or negative relationship between the rate of coverage of water and sanitation services and the growth of public expenditure in these areas. Annamraju et al. [8] recorded that, in the first decade of this century, developing countries spent between 1% and 3% of their national budgets on water and sanitation services and this was considered as inadequate to reach the millennium goals.

Although public expenditure by developing countries was classified as inadequate for the goals of universal access to water and sanitation services [8], UNWater [7] had registered a growth of 4.9%, which was also identified as being insufficient to achieve the SDGs. For example, according to UNICEF [9], in 2019 Mozambique had allocated 2.5% of the annual state budget to the water and sanitation sector, representing 0.5% of its gross domestic product (GDP), with the majority directed to urban infrastructure sustaining the trend of imbalance that occurs between urban versus rural coverage rates.

In this study, using data from the General State Account (2009–2021) and from the Mozambique Household Budget Surveys, we seek to understand whether the current pace of state budget allocations to the water sector still guarantee the achievement of the objective of SDG6 by 2030. Information about the actual investment and services delivered by a small private operator in the water and sanitation sector was excluded because of lack of regular data.

## Methodology and materials

Two methods were used to carry out this study: a literature review and data analysis from the Mozambique General State Account (CGE)^1^ and from the Household Budget Survey (IOF). The literature was identified on the Internet based on the search engine Google, Googleschoolar and from the Web of Science database. The search words used were: ‘water and sanitation and Sub-Saharan Africa’ and ‘Access to Water and Sanitation and Mozambique’. Data on the CGE are for the years between 2009 and 2021 and are available on the Ministry of Economy and Finance (MEF)’s website. The CGE is a government document that shows the budget and financial execution and simultaneously presents the results of each of the economic years, allowing an understanding of the distribution of public investment, that is, which sectors are absorbing the highest percentages of investment and vice versa.

In this study, the data collected refer to the distribution of public investment by the different sectors, including those considered by the government as a ‘priority’ for national development.

In addition to the CGE data, information on the ‘financial execution of the investment budget’ contained in the ‘Annual Water Sector Performance Assessment Reports’ prepared by the National Directorate of Water and Sanitation (DNAAS) of the Ministry of Public Works and Housing in 2013 was also used to 2017, which were available on the organisation’s website.

Data from the CGE and the Annual Water Sector Performance Assessment Reports are useful to understand the origin, evolution and trends of investment in this sector. Data on access to water and sanitation were extracted from the IOF (Table 1). The IOF (formerly known as Household Survey – IAF)^2^, which is one of the oldest data surveys carried out by the National Institute of Statistics (INE) – a government agency – is defined as a continuous and integrated survey of households, by random stratified sampling that collects using interviews, as well as sociodemographic and expenditure and income data of households residing in the country.^3^ The objective of the IOF is to obtain information on the nature and destination of consumer expenditure, as well as information about various resources related to the living conditions of households. The data is collected by all administrative units, in rural and urban areas of the country and the sample source is the national census and must be comparable between different IOFs [11,12]. The main purpose of the IOF is to support a formulation of sectoral policies and programmes for the government, private sector and civil society in general [11,12].

**Table 1.** IAFs/IOFs carried out and the number of households covered

	IAF 1996/07	IOF 2002/03	IOF 2008/09	IOF 2014/15	IOF 2019/20
Number of households interviewed	8289	8727	10,832	11,592	13,656

Source: prepared by the author (2022) based on data from IOFs (1996/07, 2002/03, 2008/09, 2014/15 and 2019/20).

In this study all the data collected in IOFs reports, CGE and in the Annual State Budget (OGE) are presented in tables and comparisons (evolution of the access to water and sanitation services between the various IOFs and tendencies of the public investment for water and sanitation from the CGE) are made to answer the question that this study proposes to investigate: can Mozambique guarantee access to water and sanitation for all by 2030 at the current pace of state budget allocations for the water sector?

In the following section, as a way of looking for answers to the formulated question, the rates of access to water and sanitation services are first compared and then the trends in investment expenditure of CGE in these two domains are analysed.

## Investment expenditure made in the water sector and sanitation services in Mozambique

Water and sanitation occupy a central place in the development policies of the government of Mozambique. The government’s Five-Year Plans (PQG) are the main planning instruments used to promote the country’s socio-economic development and are prepared with the participation of various governance institutions at different territorial scales. Every five years, and following the electoral calendar, PQGs are drawn up. These plans are executed with national, provincial and territorial annual Economic and Social Plans (PES) and the OGE is the main instrument of action and intervention that guarantees the achievement of the objectives of the PQGs. All areas of state responsibility and intervention receive annual budget allocations, which are not uniform but vary depending on national priorities. Priority areas are those that receive the largest budget allocations and are defined in the PQG. Sectors such as education, health, water and sanitation always have priority in the allocation of the annual budget.

Long before the formulation of the millennium development goals and the SDGs, access to water and sanitation has been positioned within the ‘infrastructure’ area in the CGE in Mozambique. As this is an area identified as a priority in the national development plans, the government has invested and continues to invest in increasing the coverage rate and in associated infrastructures that are considered critical. The IOFs data are useful because this allow us to capture the progress achieved in the water and sanitation services.

As a signatory to the Sustainable Development Agenda, Mozambique has formulated the following targets for SDG6: (i) to ‘achieve universal and equitable access to safe and affordable water for all by 2030’; and (ii) to ‘achieve access to adequate and equitable sanitation and hygiene for all and eliminate the practice of open defecation, with special attention to the needs of women and girls and those in vulnerable situations by 2030’.

**Figure 1:**
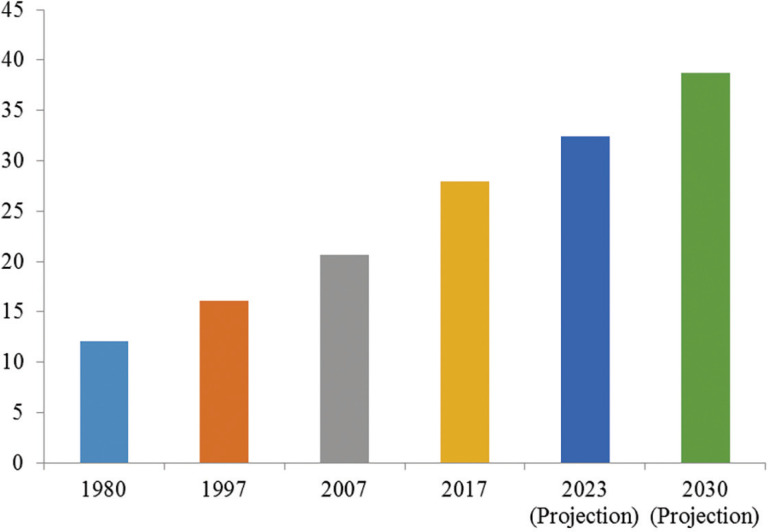
Mozambique total population by census year and projection (in million). Prepared by the author (2022) based on data from INE.

Mozambique’s population continues to grow at high rates. The national census carried out in 2007 indicated a growth of around 32.4% in relation to the previous census (1997), and around five million inhabitants had been added (one-third of the total population recorded in the 1997 census). This growth pattern was also observed with the last census (2017) but with an even faster growth rate (35%), adding just over seven million individuals to the country’s total population registered by the 2007 census (Fig. 1). The projections show that in the years to come, the total Mozambique population will continue to increase, imposing challenges in the provision of water and sanitation services to all.

**Figure 2:**
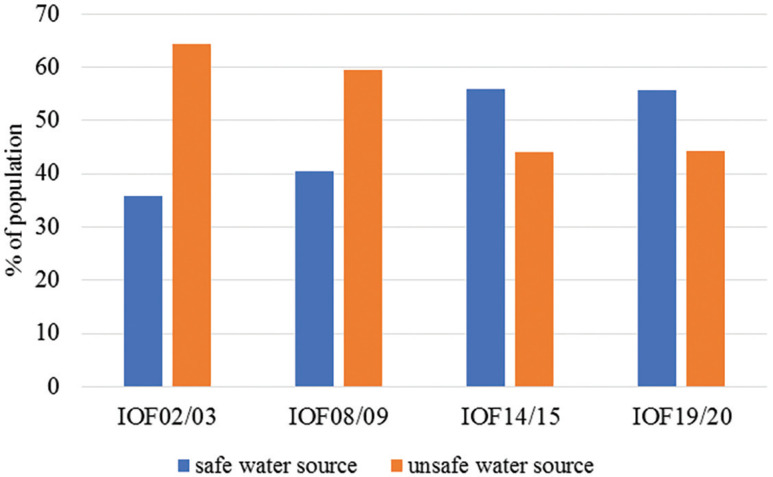
Percentage of population with access to safe and unsafe water sources, according to IOFs. Prepared by the author (2022) based on data from IOFs (2002/03, 2008/09, 2014/15 and 2019/20).

Despite the advances made, national statistics indicate that, in relation to water, the coverage rate is increasing at a fast pace, but is slower in sanitation services, that is, more than half of the total population does not have access to sanitation services. The statistics generated by the IOFs show that in 2002/03 more than half (64.2%) of the country’s population used water for consumption from an unsafe source (water from an unprotected well, spring water, water from a river, lake, pond, rainwater). This feature has been reversed; currently, more people have gained access to water from a safe source and this progress can be seen by the data of the latest IOF (2019/20), which shows that 55.7% of the population has access to a safe source of water (piped water, water from the fountain/public tap, water from the borehole/well with a hand pump, water from a protected well without a hand pump) and 44.3% continue to use unsafe water sources (Fig. 2).

When analyzing the growth pattern of the water coverage rate in Mozambique, two scenarios emerge: in the first, from 2002 (IOF 2002/03) to 2014 (IOF 2014/15), the growth observed between each of the surveys carried out was approximately 5%. In the second scenario, which coincided with the announcement of the SDGs in 2015, between the two surveys carried out (IOF 2014/15 and IOF 2019/20) the growth of the coverage rate was much slower, that is, a few tenths, very far from the 1 percentage point expected [11–14]. This pattern of low coverage can be explained by the low average public investment in access to water (see Table 2).

**Table 2.** Relationship between investments made in the water and sanitation sector (in %/year and period)

Indicators	Year												
	2009	2010	2011	2012	2013	2014	2015	2016	2017	2018	2019	2020	2021
Public investment in the water and sanitation sector (%)	3.9	4.7	4.8	4.7	4	1.3	1.5	3.8	1.7	3.6	3.1	1.1	1.1
Access to water (%)	40.5% (IOF 2008/09)					55.9% (IOF 2014/15)					55.7% (IOF 2019/20)				
Access to sanitation in % (IOF)	16% (IOF 2008/09)					29.6% (IOF 2014/15)					31% (IOF 2019/20)		
Average public investment in each period (%)	4.42						2.65						
Difference in water access rates between IOFs 2008/09–2014/15 and 2014/15–2019/20 (%)	15.4						−0.2						
Difference in sanitation access rates between IOFs 2008/09–2014/15 and 2014/15–2019/20 (%)	13.6						1.4						

Source: prepared by the author (2022) based on data from the CGE and IOFs.

**Figure 3:**
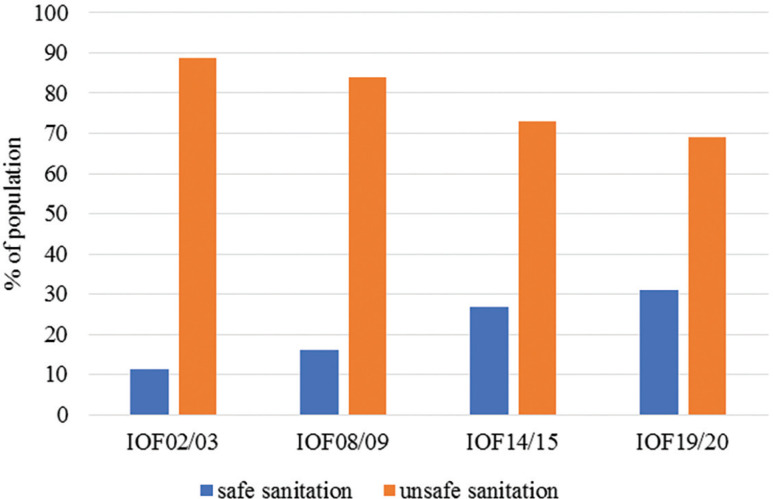
Percentage of population with access to safe and unsafe sanitation, according to IOFs. Prepared by the author (2022) based on data from IOFs (2002/03, 2008/09, 2014/15 and 2019/20).

In sanitation, access to services has made very slow progress and one of the reasons may be associated with the fact that Mozambique has unplanned informal settlements and a dispersed population in rural areas. Data from the IOF (2002/03) indicated that only 11.2% of the population had access to safe sanitation services (in-house flush toilet, outside flush toilet, non-flush toilet, improved latrine and improved traditional latrine). The most recent data generated by the IOF 2019/20 indicate that about 31% of the population has access to a safe sanitation service (Fig. 3). This is one of the fastest-growing percentages in recent years, at approximately 5% (from 2014/15 to 2019/20) [12–14].

Is it possible to find evidence that explains the trends in water coverage rates and sanitation services in the CGE? It is evident that access to water improves with new household connections, construction and rehabilitation of standpipes and small water supply systems, while access to sanitation increases with the construction of improved latrines and improved traditional latrines in rural and urban areas with connections to sewers and septic tanks. Water and sanitation services are public requirements, and in Mozambique the government has a responsibility to guarantee access to these services. In the water and sanitation sector, although the private sector can also be involved, this mission is the responsibility of three state agencies, namely the AIAS,^4^ FIPAG^5^ and DNAAS.

The analysis carried out on the budget allocations allows us to obtain an idea of the trend of public investment in the water and sanitation sector and to formulate a possible answer to the question posed. The reports produced by the DNAAS show that from 2009 to 2017 more than 70% of the investment budget came from sources outside the state budget (contributions from cooperation partners from various countries and multilateral financial organisations such as the World Bank) [16–21]. The data from the CGE show that 91% of the investment in water and sanitation services in Mozambique in 2017 came from cooperation partners (in credit and other mechanisms).

**Figure 4:**
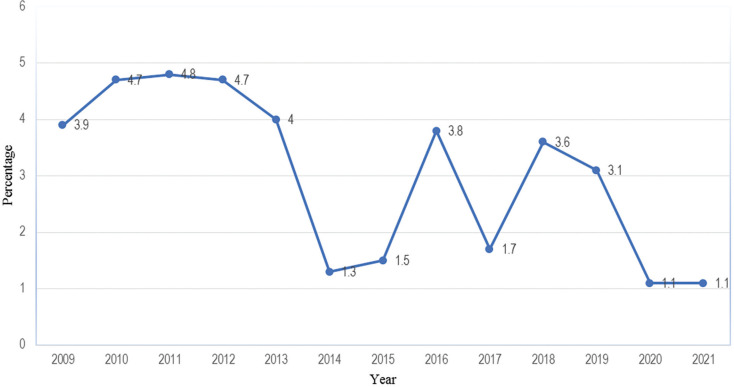
Transfers from the State Budget to the water and sanitation sector between 2009-2021 (%). Source: prepared by the author (2022) based on data from the CGE (2009-2021).

Based on the CGE, it is noted that public investment for the expansion of water and sanitation infrastructure to increase the coverage rate is very variable and inconsistent, that is, analysis of the data indicates that between 2009 and 2013 this area absorbed an average of 4% of total public investment. During these years, a constant and balanced pattern of transfers from the state to the water and sanitation sector was observed. On the contrary, from 2014 to 2021 the average was lower, at around 3% of total public investment, and with minimum percentage values reaching only 1.1%. This last period was inconsistent and with very sharp fluctuations, for example, in 2016 the allocations to the sector reached a maximum value of 3.8% of total public investment and in the following year there was a sharp reduction to 1.1%. More recently, in the latest state budgets (2020 and 2021) the percentage allocated to the sector was 1.1%, which is the lowest value observed in the entire period of this analysis (Fig. 4) [22–31].

There is a pattern that emerges which shows a positive relationship between the increase in access to water and sanitation with the state transfers for investment in water. This increase was sometimes very noticeable and other times very low. The absolute and relative values of the CGE show that:

1. between 2009 and 2013, the volume of investment in this sector was the highest recorded until 2021 (Fig. 4). The effects of this investment can probably explain the reduction in the population without access to a safe water source and the increase in the population with access to sanitation services that was captured by the IOF 2014/15;
2. the slow growth in the water and sanitation coverage rate that occurred after 2015 is somehow related to the investment made in that period, which was very reduced when compared to the previous period (2009–2013); and
3. the years between 2014 and 2020 were those with inconstant public investment and frequent fluctuations, with the lowest minimum values observed throughout the period (2009–2021) and it is also the period in which the water and sanitation coverage rate grew very little.

## Discussion and conclusion

Here the main results are discussed. The discussion is based on a combination of indicators that make it possible to establish the relationship between public investment in the water and sanitation sectors from 2009 to 2021 with the rate of access to these services, and also to project from the trends of current investment to predict whether it will be possible to reach the water and sanitation SDG target in 2030.

Table 2 summarises the results of this study. With it, it is possible to observe that the average value of public investment carried out in the period between 2009 and 2013 was 4.42%. In this same period, the average rate of water coverage was 15.4% and access to sanitation services was 13.6%. This rate is the highest recorded to date. In contrast, in the period between 2015 and 2018 the average value of public investment in these two domains was 2.65%. During this period, the water coverage rate was negative (−0.2%), that is, it did not grow, despite the increase in the country’s total population. From 2020 to 2021, investment in the water and sanitation sector was around 1.1%, which is the lowest value in the entire period (2009–2021). If this last percentage does not increase, the access rate will continue to be negative, that is, a large part of the population will be without access to water and sanitation in 2030. The data in this study shows that when investment increases, access to water and sanitation also increases.

The data presented in this table show that if the average investment observed in the period 2009–2013 of about 4.42% had been maintained in the period between 2015 and 2018, access to safe water sources would have reached 71% of the population, and if the same investment value were held at least constant for the years ahead, Mozambique could reach the target of access to water for all one year earlier than the date set by the UN. In the case of sanitation, if the country maintains an average investment of 13.6%, as was observed in the period between 2009 and 2013, it will be 2043 before the entire population will have access to safe sanitation services. For all people to have access to safe water sources in Mozambique by 2030, and in order to accelerate the pace, the country must allocate 10–12% of total public investment to the water sector. This figure increases to around 20% for sanitation. This will be a big challenge for a country that has many basic services to provide for all its population.

## Data Availability

The datasets generated during and/or analysed during the current study are available in the repository: https://www.mef.gov.mz/ and https://www.ine.gov.mz.

## Notes

1 All the acronyms are in the Portuguese language. This option is to maintain some originality of the names of the institutions and national government documents.

2 Inuérito aos Agregados Familiares (IAF), in Portuguese, was the first name for the Household Budget Surveys (IOF).

3 For the IOF estimates to be representative of the population, it was necessary to multiply the data by a weighting factor. The basic weight for each selected household was calculated as the inverse of its selection probability. The basic weight, or expansion factor, is calculated as the inverse of this selection probability. The sum of the household weights is equal to the total number of households in the country and the multiplication of the average number of members in each household by the total number of households must be equal to the total population at half of the data collection period ([10]:12).

4 The Water and Sanitation Infrastructure Administration (AIAS) is the entity responsible for managing the heritage and public investment programme in the water supply and wastewater sanitation infrastructure in smaller cities, such as towns and districts outside the jurisdiction of the FIPAG [15].

5 FIPAG is the body responsible for managing public water supply services in the country’s large cities and towns. It is the entity responsible for attracting and managing investments for the development of infrastructure in Mozambique’s main cities and for promoting a strong partnership between the public and private sectors [15].
